# Inclusion of LGBTQ+ Individuals in Hong Kong: A Scoping Review

**DOI:** 10.1007/s13178-024-01003-5

**Published:** 2025-03-06

**Authors:** Eliz Miu Yin Wong, Yiu Tung Suen, Randolph C. H. Chan, Suchon Tepjan, Peter A. Newman

**Affiliations:** 1 Gender Studies Programme, The Chinese University of Hong Kong, Room 250, 2/F, Sino Building, Hong Kong, China; 2 Department of Social Work, The Chinese University of Hong Kong, Hong Kong, China; 3 VOICES-Thailand Foundation, Chiang Mai, Thailand; 4 Factor-Inwentash Faculty of Social Work, University of Toronto, Toronto, Canada

**Keywords:** LGBTQ+ inclusion, Gender and sexual minorities, Scoping review, Asia, Hong Kong

## Abstract

**Introduction:**

Discussion on the rights and inclusion of lesbian, gay, bisexual, transgender, and queer (LGBTQ+) individuals in Hong Kong is often based on taken-for-granted socio-cultural norms but not grounded in empirical studies.

**Methods:**

A scoping review was conducted to map out the current status of LGBTQ+ inclusion in Hong Kong, with results reported according to PRISMA-ScR guidelines. The review captured 1205 unduplicated studies which were scoped to 152 peer-reviewed articles published from 2010 to 2022. Findings were categorised using an adapted Global LGBTI Inclusion Index into six domains: education, economic well-being, family, health, political and civic participation, and personal security and violence.

**Results:**

LGBTQ+ individuals in Hong Kong face prevalent exclusion and discrimination across major domains of life, which does material harm to their well-being. This underscores the importance of legal protection and recognition to address the exclusion of LGBTQ+ individuals.

**Conclusions:**

Gaps were identified for future research: (a) the need to address understudied domains other than health; (b) the need to focus on understudied populations (lesbian, bisexual, and transgender people); (c) the wide range of terminologies adopted in existing LGBTQ+ research; and (d) lack of representative data.

**Policy Implications.:**

This review highlights the importance of collecting empirical data and obtaining representative data on the LGBTQ+ population to assess the current situation and progress made in LGBTQ+ inclusion in Hong Kong, and to inform policy changes related to LGBTQ+ rights.

## Introduction

Discussions of the rights and inclusion of lesbian, gay, bisexual, transgender, and queer (LGBTQ+) individuals in Asian societies are often based on assumptions and perspectives developed from taken-for-granted socio-political and cultural norms and anecdotes. They do not, however, necessarily reflect the realities faced by the community. Public debates on LGBTQ+ rights and related policies are also often clouded with personal viewpoints informed by cultural and moral values, and these arguments often lack grounding in empirical studies. Empirical research can inform policy debates and help counter stereotypes and morality-based arguments by demonstrating the tangible harm of excluding LGBTQ+ individuals, both for the community and for society as a whole (Badgett & Crehan, 2017).

There is growing interest around LGBTQ+ issues in Hong Kong. In terms of legal developments, there are a number of completed and ongoing court cases concerning LGBTQ+ rights, such as same-sex marriage, same-sex couples’ right to immigration, housing, fringe benefits, and legal gender recognition, among others (Suen, 2021; Suen et al., 2022a). However, there is a scarcity of official data on the population, limited scholarly attention, and little representation in mass media (Suen, 2017). This may be due to the invisibility and marginalisation of LGBTQ+ individuals in Hong Kong, where legal protection and recognition are limited or non-existent (Tang et al., 2020). To gain a more systematic understanding of the experiences of LGBTQ+ individuals in Hong Kong and the multisectoral impacts on their lives, we conducted a scoping review to assess the state of the empirical literature and map out the current status of LGBTQ+ inclusion in Hong Kong, with a view to inform policymaking.

### Legal and Social Context of LGBTQ+ Inclusion in Hong Kong

The understanding of LGBTQ+ inclusion in Hong Kong needs to be contextualised in the historical, cultural, and social contexts of Hong Kong society, a post-colonial city strongly influenced by Chinese culture. Same-sex sexual activity between consenting male adults was first criminalised when Hong Kong became a British colony in 1842. In 1991, same-sex sexual activity between consenting male adults over the age of 21 was decriminalised. The age of consent (over the age of 16) was equalised between heterosexuals and homosexuals in 2006 as a result of judicial review (Suen & Wong, 2017). Currently, there is no anti-discrimination ordinance in Hong Kong offering legal protection against discrimination based on sexual orientation or gender identity in the private sector (Suen & Chan, 2021). The Hong Kong Court of Final Appeal, however, has held that Hong Kong’s Basic Law and Bill of Rights Ordinance protect against discrimination based on sexual orientation. While these laws do not provide protection against discrimination in the private sector, they provide protection against discrimination in government functions, such as public housing, immigration, and taxation.

There is minimal legal recognition of same-sex relationships registered overseas, and same-sex marriages and civil partnership obtained abroad are recognised for a host of legal purposes in Hong Kong, including immigration, tax, housing, and civil service spousal benefits, as a result of judicial review decisions. In September 2023, the Hong Kong Court of Final Appeal ruled that the Hong Kong Government must establish a legal framework to recognise the rights of same-sex couples within 2 years.

There is also no system for legal gender recognition in Hong Kong. In different domains of life, the legal uncertainty around legal gender recognition still exists (Suen et al., 2022a). Although a recent court case in 2023 has ruled that "full" "sex reassignment surgery" is no longer required of transgender people for a change of the gender marker on their identity card, organisations that work with transgender people have reported to the media that the Immigration Department has not put this ruling into practice.

Under the intersecting influences of British colonial history and traditional Chinese culture, LGBTQ+ individuals in Hong Kong experience stigma and prejudice to which religion and traditional family values contribute (Suen & Wong, 2017). A certain dominant interpretation of Christianity in some mainstream churches perceives homosexuality as sinful, and this view pervades various social institutions in Hong Kong, including schools, medical facilities, and social services. Additionally, traditional Chinese culture, which emphasises family values and the importance of having offspring to carry on the family name, puts immense pressure on LGBTQ+ individuals to conform to traditional gender and family norms, get married, and have children (Kong, 2021). These factors are foundational to the widespread stigma, discrimination, and victimisation experienced by LGBTQ+ individuals in Hong Kong.

In recent years, there has been a noticeable shift in public attitudes in Hong Kong towards LGBTQ+ rights. A joint report issued in 2023 by the University of Hong Kong, the Chinese University of Hong Kong, and the University of North Carolina tracked significant growth in public acceptance of same-sex partnerships. The report highlighted that, in 2023, 60% of Hong Kong’s population supported same-sex marriage, a significant increase from only 38% in 2013. Additionally, some professional organisations have started to embrace an LGBTQ-inclusive stance and have adopted various position statements and codes of conduct. For instance, the Hong Kong Social Workers’ Registration Board (2013) implemented a Code of Conduct that includes respecting "dignity of every human being, irrespective of one’s… sexual orientation." Similarly, the Hong Kong Psychological Society (2023) promulgated a position paper promoting LGBTQ inclusivity, and the Hong Kong College of Psychiatrists (2011) publicly opposed conversion therapies. These changes provide a foundation for fostering LGBTQ+ inclusion in Hong Kong society.

### Global Inclusion of LGBTQ+ Individuals

The inclusion typology developed by Badgett and Sell (2018) is a global index that measures LGBTQ+ inclusion in terms of "access to opportunities and achievement of outcomes…as well as human development" (Badgett & Sell, 2018, p. 1). The global index aims to assess inclusion in different countries worldwide, compare the overall degree of inclusion across societies, and measure progress towards inclusion over time. The typology highlights five social domains that are essential to the full inclusion of the LGBTQ+ population: education, economic well-being, health, political and civic participation, and personal security and violence. These five domains are perceived to cover the most important aspects of LGBTQ+ life. However, as will be illustrated in this scoping review, existing literature on Hong Kong points out the centrality of family in shaping the lives of LGBTQ+ individuals. Under the strong influence of Confucianism, family is perceived as the major heteronormative regulatory force for LGBTQ+ individuals in Hong Kong (Kong, 2011). We suggest that there is a need to localise the typology when it is applied to different cultural contexts. In the case of Hong Kong, we argue that it is important to include the family domain in the inclusion typology, while it may also well be the case that other domains need to be included in the typology in other societies.

###  The Present Study

This scoping review explores the breadth and depth of existing literature on LGBTQ+ inclusion in Hong Kong. It also synthesises empirical evidence of the current lived experiences of LGBTQ+ individuals across multiple domains, identifies knowledge gaps, and provides recommendations for future research and policy directions. The purpose of this study was to map the literature on evolving topics regarding LGBTQ+ inclusion in Hong Kong and identify gaps. We therefore chose to conduct a scoping review rather than a systematic review, which typically focuses on the feasibility or effectiveness of a specific practice (Munn et al., 2018).

## Methods

We adopted the scoping review framework initially proposed by Arksey and O’Malley (2005) and further developed by the Joanna Briggs Institute (2015). The findings are reported in accordance with PRISMA-ScR guidelines (Tricco et al., 2018). The key steps were (a) identify research questions, (b) define a search strategy, (c) establish a set of inclusion and exclusion criteria, (d) study the selection based on the inclusion and exclusion criteria, (e) chart and summarise the data, and (f) report the results.

### Research Question

The scoping review was guided by the following research question: How does the existing literature describe LGBTQ+ inclusion in Hong Kong in the following six areas—education, employment, family, health, personal security and violence, and political and civil participation?

### Information Sources and Search Strategy

A literature search was conducted in the following databases: EBSCO (Bibliography of Asian Studies, Child and Adolescent Development, Econ Lit, Education Source, Gender Studies, LGBT Life); HeinOnline Hong Kong; OVID (Medline, PsychINFO Search String); and ProQuest (Applied Social Sciences Index and Abstract, Dissertations and Thesis, ERIC, Intl Bibliography of the Social Sciences, PAIS Index, Policy File Index, PsychARTICLES, Worldwide Political Science Abstracts). To conduct these searches, we used search strings that have previously been validated for the LGBTQ+ population (Lee et al., 2016). We added relevant Hong Kong LGBTQ+ terminology, including "Tongzhi" and "Memba." "Tongzhi" is a term commonly used to denote individuals with non-normative gender and sexualities in Chinese society (Kong, 2011; Lau et al., 2017), while "Memba" is local term used for self-identification among gay men in Hong Kong (Kong, 2011). We also included the term ‘Hong Kong’ in the search strings to limit the search results geographically. Table 1 lists the search strings used in this scoping review.

**Table 1:** Search strings used for literature search

**Location:**	
1. **Hong Kong**	
2. **Hong Kong Special Administrative Region of the People’s Republic of China**	
3. **HKSAR**	
**LGBTQ+ terminologies**	
1. Bicurious 2. Bisexual 3. Bisexuality 4. Bisexuals 5. Cross sex 6. Crossgender 7. F2M 8. Female-to-male 9. Gay 10. Gays 11. Gender change 12. Gender dysphoria 13. Gender identity 14. Gender queer 15. Gender reassign 16. Gender transform 17. Gender transition 18. Genderqueer 19. GLB 20. GLBQ 21. GLBs 22. GLBT 23. GLBTQ 24. Heteroflexible 25. Homosexual 26. Homosexualities 27. Homosexuality 28. Homosexuals 29. Intersex 30. Lesbian 31. Lesbianism 32. Lesbians 33. Lesbigay 34. LGB 35. LGBQ 36. LGBS 37. LGBT 38. M2F 39. Male-to-female 40. **Memba** 41. Men who have sex with men	42. MSM 43. Queer 44. Same gender loving 45. Same sex attracted 46. Same sex couple 47. Same sex couples 48. Same sex relations 49. Sex change 50. Sex reassign 51. Sex reversal 52. Sex transform 53. Sex transition 54. Sexual and gender minorities 55. Sexual and gender minority 56. Sexual identity 57. Sexual minorities 58. Sexual minority 59. Sexual orientation 60. Sexual preference 61. **Tongzhi** 62. Trans female 63. Trans male 64. Trans man 65. Trans men 66. Trans people 67. Trans person 68. Trans woman 69. Trans-sexuality 70. Transexual 71. Transgender 72. Transgendered 73. Transgenders 74. Transsexual 75. Transsexualism 76. Transsexuality 77. Transsexuals 78. Transvestite 79. Women loving women 80. Women who have sex with women 81. WSW

Search strings used were based on Lee et al. (2016)’s article. Added terminologies are shown in bold

### Study Selection Criteria

This scoping review included peer-reviewed articles published between 1 January 2010 and 31 December 2022. As the first synthesis of published evidence of research on LGBTQ+ people in Hong Kong, we were interested in empirical studies conducted within the last 12 years, as these are most relevant to current policies and law. The rationale for choosing 2010 as the starting date was based on the context of the development of LGBTQ+ rights in Hong Kong. Kong et al. (2015) divided the Hong Kong Tongzhi movement since 1979 into four waves, each emerging anew but retaining elements of the previous waves. They noted that the third wave spanned from 1997 to the 2000s; it was characterised by the construction of citizenship and the emergence of the politics of difference. The fourth wave is ongoing development that is marked by increased queer visibility and the potential for rights advocacy. Based on the analysis of Kong et al. (2015), this scoping review took 2010 as its starting point to capture empirical studies conducted during the fourth wave of LGBTQ+ inclusion in Hong Kong.

The inclusion criteria for this review were as follows: (a) a focus on LGBTQ+ individuals or communities; (b) a focus on people residing in Hong Kong (including refugees and/or non-citizens); and (c) research data on inclusion, marginalisation, discrimination, disparities, human rights, or well-being of the LGBTQ+ population.

The publications were excluded if they (a) were published before the year 2010; (b) were not written in English or Chinese; (c) did not focus on LGBTQ+ people or communities; (d) did not focus on individuals residing in Hong Kong (e.g. Hong Kong people living abroad); (e) were a review or meta-analysis; and (f) did not include primary or empirical data or analysis.

### Study Selection Process

The searches from the databases mentioned above were documented and imported into Covidence, a software program for systematic reviews, to manage abstract and full-text screening. Two reviewers independently screened titles and abstracts for inclusion. All conflicting votes were resolved by the third reviewer. Two reviewers then read the full text of potentially relevant articles and determined whether the articles fulfilled the pre-set inclusion criteria. All discrepancies between reviewers were resolved by the third reviewer. The interrater reliability was 88% (Cohen’s kappa = 0.63, substantial level of agreement) at the abstract review stage, and 84% (Cohen’s kappa = 0.63, substantial level of agreement) at the full-text review stage (Landis & Koch, 1977).

### Data Extraction and Synthesis

Data on publication characteristics were extracted, including publication year, author(s), research methods (i.e. qualitative, quantitative, or mixed methods), focal population(s), sample size, and key study findings about LGBTQ+ inclusion. We analysed the findings quantitatively (i.e. research methods and targeted population) and qualitatively (i.e. thematic analysis) to determine the main domains of LGBTQ+ inclusion addressed. Three of the authors identified and categorised the publications into six key domains, and any discrepancies were discussed and resolved by consensus in research team meetings.

## Results

**Fig. 1:**
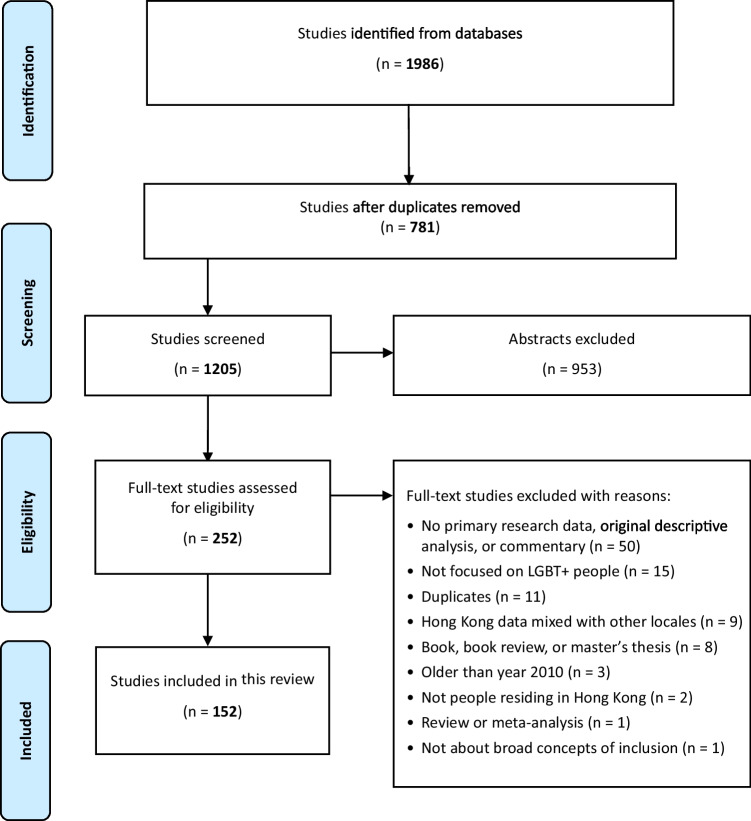
PRISMA flow diagram of study selection process and results Search strings used were based on Lee et al.’s (2016) article. Added terminologies are shown in bold

The search yielded 1205 articles after removing duplicates. Screening of titles and abstracts resulted in 252 articles included in the full-text review. After full-text screening based on the a priori inclusion and exclusion criteria, we identified 152 peer-reviewed articles for inclusion in the scoping review. Figure 1 illustrates the study selection process.

Among all included articles (*n* = 152), 68% (104) were quantitative studies, 31% (47) were qualitative studies, and 1% (1) was a mixed-method study. The coverage of the topic of LGBTQ+ inclusion has increased in peer-reviewed articles over the past 12 years. Most of these articles (67.8%, *n* = 103) were published between 2017 and 2022, which is the second half of the scoping period. In general, we use the term LGBTQ+ to refer to the overall picture in Hong Kong, but we also use other specific acronyms to reflect the focal populations and findings of a particular study more accurately. Fig.1 PRISMA Flowchart of Study Selection

### Study Characteristics

A wide variety of terminology for focal populations was used among the peer-reviewed articles. One-quarter (26%; 39) adopted an identity-based approach to identify their focal population and focused on specific subgroups; 8% (12) adopted the term gay men, while 3% (4) of the articles focused on gay and bisexual men. Similarly, 5% (7) of the articles focused on lesbians, while 2% (3) focused on lesbian and bisexual women, and 3% (4) focused on gay men and lesbian women. One article (1%) adopted the term "homosexuals." Two papers focused specifically on bisexual and pansexual individuals (1%). For the transgender community, one article (1%) used the term ‘trans*’, while another four papers (3%) adopted the term ‘transgender’. One paper (1%) focused on transgender and genderqueer people.

Another 22% (34) of the articles adopted an identity-based approach, but addressed LGBTQ+ people as a group, with different terminologies: 8% (12) of the articles focused on LGB, while 2% (3) included LGBQ and 3% (4) of the articles adopted the term LGBT. Overall, 1% (2) of the studies used the term ‘non-heterosexual’ and 7% (10) adopted the term ‘sexual minorities’. Two articles (1%) used the term "sexual orientation minorities," while another article (1%) used the term "sexual and gender minorities."

Overall, 37% (56) of the articles adopted a behavioural approach to identify their focal population; 36% (54) of the articles used the term men who have sex with men (MSM). These articles mainly focus on sexual health. Another 1% (2) of the studies focused on respondents who have/had same-sex experiences or same-sex romantic partners. Another 3% (5) of the articles focused on MSM who are living with HIV. Other articles focused on respondents with certain affiliations, such as members of LGBT organisations (1%, 2), members of pro-LGBT Christian organisations (1%, 1), and migrant domestic workers (2%, 3).

Thirteen articles (9%) focused on the general public’s attitudes towards the LGBTQ+ community and other stakeholders closely associated with LGBTQ+ individuals; 3% (4) of the articles addressed public attitudes towards the LGBTQ+ community. Other studies focused on parents of LGB children (1%, 1), secondary students (1%, 1), social workers (1%, 1), social work students (1%, 2), teachers (1%, 1), and blood donors (1%, 2).

### Research Domains

Among the six domains of LGBTQ+ inclusion, most studies focused on health (72%, 110). Others focused on political and civic participation (13%, 20), economic well-being (8%, 12), education (7%, 11), and personal security and violence (3%, 5). One-fifth (20%, 30) of the studies focused on more than one of these areas. However, 18% (27) of the studies focused on family, a domain that Badgett and Sell (2018) did not include in their typology.

#### Health

Among the 72% of articles (110) focused on health, more than half (53%, 59) addressed sexual health, particularly HIV/STI transmission and prevention. The second most prevalent focus, with 42% of the articles (47), was mental health within the LGBTQ+ community. Only 3% (3) addressed physical health outside of HIV/STIs, although 5% (6) of the articles focused on the impact of the COVID-19 pandemic on the LGBTQ+ community.

##### Sexual Health

Among articles addressing sexual health, most focused on HIV-related topics among MSM (45% of the papers in the health domain). Thirty HIV-related publications addressed the risk and protective factors of safer sex practices, mainly focusing on condom use in anal sex (Cai & Lau, 2014; Choi et al., 2021; Kwan et al., 2020; Lau et al., 2013a, 2014b, 2016; Leung et al., 2015; Li et al., 2014; Liang et al., 2015; Poon & Lee, 2013; Tsui et al., 2014; Wang et al., 2020c; Wong et al., 2020; Yang et al., 2022; Yeo & Fung, 2016) and PrEP use (Kwan et al., 2021; Kwan & Lee, 2018; Lau et al., 2022; Suen et al., 2022b; Wang et al., 2018a, 2020b, 2021b; Wong et al., 2018). Risk factors for unprotected anal sex included chemsex (recreational drug usage during sex; Kwan et al., 2020; Wang et al., 2020c; Wong et al., 2020), as well as cultural beliefs that condomless anal sex and internal ejaculation are symbols of trust and commitment with regular partners (Yeo & Fung, 2016). During sexual activity, situational factors such as discussing condom use with partners and environmental variables like the availability of condoms can serve as protective factors for condom use (Lau et al., 2016; Li et al., 2014). Despite high acceptance (Kwan & Lee, 2018) and prevention-effective adherence to either daily or on-demand PrEP (Kwan et al., 2021), affordability (Lau et al., 2022) and PrEP-related stigma (Lau et al., 2022; Suen et al., 2022b) were anticipated challenges in promoting PrEP uptake in Hong Kong. PrEP users in Hong Kong can only get a prescription from registered private doctors or overseas doctors (e.g. from Thailand, among users commonly known as ‘PrEP tourists’; Suen et al., 2022b; Wang et al., 2021b).

Eleven studies examined willingness and uptake of voluntary counselling and testing (Gu et al., 2011; Lau et al., 2013b), as well as HIV self-testing (Chan et al., 2021; Kwan et al., 2022b; Wang et al., 2018b; Wong et al., 2015). The perceived discrimination and perceived empathy of service providers were associated with HIV re-testing intention (Gu et al., 2015). Low usage and acceptability rates on self-testing were found, despite its availability in the market (Wong et al., 2015). Promotional and intervention programmes have also been developed to promote HIV self-testing (Suen et al., 2022b; Wang et al., 2021b).

Only five articles focused on MSM living with HIV (Chan, 2018b; Chong et al., 2017; Kwan et al., 2022a; Lee et al., 2018; Poon et al., 2018). A low level of sexual risk behaviours was found among MSM living with HIV, which indicates a general reduction in secondary transmission (Poon et al., 2018). However, drug use behaviours have been reported among MSM living with HIV (Lee et al., 2018). In addition, HIV stigma among MSM living with HIV has negative impacts on their mental health (Chan, 2018b; Chong et al., 2017). Despite low awareness, positive attitudes towards functional cure and willingness to participate in related clinical trials were found among MSM living with HIV (Kwan et al., 2022a). Three articles discussed the blood donation practices in Hong Kong (Lau et al., 2021; Lee et al., 2013; Lee & Lee, 2014). In 2017, the policy changed from a life ban to a 12-month deferral for HIV screening for MSM to donate blood. A high level of acceptance of this time-limited deferral among blood donors was found (Lau et al., 2021).

Other than HIV-related studies, six articles focused on HPV prevention, including vaccine uptake (Chan et al., 2022a; Lau et al., 2013c; Wang et al., 2013; Wang et al., 2021a, c) and testing (Chan et al., 2022a). The perceived susceptibility and perceived severity of HPV and HPV-related diseases were associated with HPV vaccine uptake (Lau et al., 2013c; Wang et al., 2013, 2021c). Acceptability of HPV vaccines was highly price sensitive (Lau et al., 2013c), while HPV screening alone is not preferred among MSM (Chan et al., 2022a). Two articles focused on the hepatitis C virus (HCV) and HCV testing uptake (Kwan et al., 2022c; Wang et al., 2020a).

The vast majority of HIV-related studies focused on MSM. Only one article focused on transgender people in Hong Kong and revealed they have a low level of consistent condom use during sex and low HIV-testing uptake (Suen & Chan, 2020).

##### Mental Health

The 47 articles that addressed the mental health of LGBTQ+ individuals in Hong Kong identified numerous challenges, including self-stigma, internalised homonegativity, sexual orientation concealment, perceived unfriendly environments, experienced and anticipated discrimination, and sexual orientation victimisation. These factors have been found to have a negative impact on LGBTQ+ mental health (Chan, 2018a; Cheung & Chan, 2021; Hu et al., 2022; Huang & Chan, 2022; Lo et al., 2022; Mak et al., 2010). Additionally, some LGBTQ+ individuals may feel compelled to undergo sexual orientation change efforts, a scientifically discredited practice commonly known as ‘conversion therapy’, which increases their risk of developing depressive symptoms and suicidal ideation (Chan et al., 2022b).

Protective factors for LGBTQ+ individuals’ well-being have also been identified. At the individual level, mindfulness and self-compassion were associated with lower levels of self-stigma, depressive symptoms, and anxiety (Chan et al., 2020a; Chan & Leung, 2021; Lau et al., 2020). Social support and perceived family LGBT-friendliness could help reduce the harm of sexual orientation concealment and discrimination (Chen & Hung, 2021; Huang & Chan, 2022). Developing community connections and group identification could also help build resilience against stigma, develop a positive sexual identity, develop self-empowerment, and improve life satisfaction (Yip & Chan, 2022). A sense of community was found to mobilise LGB individuals to take individual and collective action (Yip & Chan, 2021). Private collective action against heterosexism, such as correcting people when they use heterosexist language, has also been found to moderate the association between perceived discrimination and depressive symptoms among sexual minorities (Chan & Mak, 2021).

Most studies on mental health focused on sexual minorities as a general group, with only two articles focusing specifically on bisexual/pansexual individuals in Hong Kong. Bisexual individuals were more likely to report identity uncertainty, experience sexual orientation concealment, and have a weaker sense of connection to the LGBT community, which are in turn associated with higher levels of depressive and anxiety symptoms than lesbians and gay men (Chan et al., 2020b). Monosexism discrimination experienced by bisexual/pansexual individuals has also been associated with higher levels of depression and anxiety symptoms, above and beyond heterosexist discrimination (Chan & Leung, 2023). Only one article examined the mental health of transgender individuals in Hong Kong. It reported that 67.0% of 106 transgender respondents had contemplated suicide, and 20.8% had attempted suicide (Suen et al., 2018). Transgender individuals in Hong Kong are subject to widespread discrimination, with very limited legal protections and social support.

##### Physical Health

Only three articles identified were related to physical health. One study found experienced and anticipated discrimination among LGB individuals to be associated with greater sleep disturbance and poorer physical health (Chan & Fung, 2021). Another study with over 3000 secondary students found that gay and bisexual boys reported poorer physical health than their heterosexual peers (Zhang et al., 2017). Finally, one article addressed the heterogeneity among transgender people in their desire to undergo gender-affirming medical interventions based on various concerns, such as financial constraints and reservations about surgical risks and/or techniques (Suen et al., 2022a).

##### Access to Social and Medical Services

Four studies addressed access to services and indicated that sexual minorities may experience difficulties getting support from medical and social services. The intention to seek professional mental health support among MSM who are at risk of mental health issues was associated with the perceived empathy of mental health service providers and their willingness to disclose their sexualities (Mo et al., 2018). However, due to the unfriendly attitudes of general society and healthcare providers in Hong Kong towards sexual minorities, MSM found it difficult to feel safe coming out and seeking help from professionals. A study conducted with social work students showed that those with Christian beliefs tended to hold negative attitudes towards lesbians and gay men (Kwok et al., 2013). Moreover, social workers may not have sufficient training on sexual and gender diversity, as it is neither common nor compulsory in social work training programmes (Kwok et al., 2013). Due to the prevalence of prejudice against sexual minorities and the fear of facing oppression from heteronormative organisations, social workers may even feel unsafe discussing sexual diversity information and providing support for LGBQ individuals (Kwok, 2021).

##### COVID-19

Six articles addressed the impacts of the pandemic on LGBTQ+ individuals. These studies covered topics such as well-being, changes in sexual behaviour, access to HIV services, and the prevalence of COVID vaccination uptake. LGB individuals were particularly vulnerable to poor mental health during the pandemic. COVID-19-related stressors specific to sexual minorities, such as frequent family conflict related to sexual orientation and a reduced connection to the LGBT+ community, have been shown to contribute significantly to depressive and anxiety symptoms beyond general COVID-19-related stressors (Suen et al., 2020). For those living with parents during the pandemic, parental unaccepting attitudes have been linked to increased family conflict, which in turn was associated with higher levels of loneliness (Suen et al., 2023). Notably, the experiences of LGB individuals are intersectional and related to living arrangements and relationship status (Suen et al., 2022c). Some LGB individuals have reported positive impacts, such as having more time and space with their partners and connecting with the LGBTQ+ community online during the pandemic (Suen et al., 2022c). One study examined the motivations, perceived difficulties, and risks of having sex among gay and bisexual men during the pandemic (Suen et al., 2021b). Perceived difficulties in accessing HIV services have also been observed during the pandemic (Suen et al., 2021a). Another study found that perceived susceptibility and severity, both general and MSM-specific, were positively associated with COVID-19 vaccination behaviour (Yu et al., 2022).

#### Family

A total of 27 articles (18%) focused on the families of LGBTQ+ individuals in Hong Kong. Of these, 74% (20) focused on LGBTQ+ individuals’ relationships with their family of origin, while 44% (12) discuss their rights to marry and form a family. Extensive studies found that LGBTQ+ individuals in Hong Kong face pressure from their families of origin (Kong, 2020, 2021; Tang, 2022). Under the influence of filial piety, LGBTQ+ individuals are expected to conform to familial heteronormativity and obey their parents’ wishes for marriage and producing offspring to continue the patrilineal line (Kong, 2021; Tang, 2021). LGBTQ+ individuals often find it difficult to come out to their parents and face challenges gaining their acceptance. A study of 1457 LGBQ+ individuals in Hong Kong found that the parents of nearly half of them did not accept their sexual orientation (42.3% and 48.6% had unaccepting mothers and fathers, respectively; Suen et al., 2023). Some LGBTQ+ individuals have undergone sexual orientation change efforts, pseudoscientific practices commonly known as ‘conversion therapy’, because they were advised to do so by their family members or wanted to face less family rejection (Chan et al., 2022b). A study with parents of LGB children showed that parents’ unaccepting attitudes towards their child’s sexual orientation have a detrimental effect on their own well-being (Chan, et al., 2022c).

Generational differences in family relationships among gay men, lesbians, and bisexual women in Hong Kong have been found. Studies with older gay men showed that they face strong pressure to marry, which leads to a double life. They feel obligated to be a filial son, loyal husband, and stern father, while also exploring their same-sex romantic relationships outside their families (Kong, 2012). Similar experiences have been found in older lesbian and bisexual women (Tang, 2022). Early marriage was common, and older lesbian and bisexual women fulfilled their family obligations to support their families (Tang, 2021). In the younger generation, gay men in Hong Kong define their achievements by gaining a good education and undertaking a respectable career. Some of them live openly as gay men and participate in gay communities. Their parents’ reactions range from total opposition to full acceptance, while silent tolerance is among the most common reactions (Kong, 2020). However, their families’ unaccepting attitudes and exclusion create a social barrier for them to advocate and claim their rights (Chan, 2017).

Twelve articles focused on LGBTQ+ rights to marriage and family formation. At present, Hong Kong only legally recognises same-sex marriage or civil partnerships registered overseas in some limited domains (Suen & Chan, 2021; Tang et al., 2020). In September 2023, the Hong Kong Court of Final Appeal ruled that the Hong Kong Government must establish a legal framework to recognise the rights of same-sex couples within 2 years. However, the details and the extent of recognition that this legal framework would provide remain uncertain. The lack of recognition has affected LGBTQ+ individuals’ current well-being and future plans.

Nearly half of LGB respondents (48.0%) in one survey (*n* = 920) considered leaving Hong Kong because same-sex marriage is not legalised, and 26.0% considered leaving Hong Kong due to the difficulties same-sex couples face in having children (Suen & Chan, 2021). Lesbian and gay men in midlife indicated a heightened sense of insecurity and uncertainty about ageing and future life plans due to the lack of legal recognition of same-sex relationships (Lo et al., 2022). Anxiety about future care and end-of-life concerns were also shared by ageing non-heterosexual migrants in Hong Kong (Suen, 2022).

Same-sex couples situated their same-sex unions in a larger family-kinship system while attempting to meet the moral responsibility of filial piety (Tang et al., 2020). A study with Chinese lesbians (*n* = 438) suggested that most of them (92%) supported legalising same-sex couples’ access to assisted reproductive technology, but less than half (41%) wanted to use it to have their own children (Lo et al., 2016).

A few studies explored same-sex relationships beyond the scope of marriage rights and adoption. Two articles delved into the domestic labour dynamics among lesbian couples and the process of meaning-making among those who consider themselves as families, beyond the marriage institution (Wong, 2012, 2013). Intimate partner violence (IPV) within same-sex relationships was found to be prevalent (Chong et al., 2013; Mak et al., 2010). In a study with 339 LGB participants, 79.1% reported experiencing IPV at least once, with almost half experiencing multiple forms of abuse, including psychological, physical, or sexual abuse (Mak et al., 2010).

#### Political and Civic Participation

Twenty articles (13%) explored the political and civic participation of LGBTQ+ individuals in Hong Kong. In the face of the absence of legal protection against discrimination and the lack of legal recognition for same-sex relationships, LGBTQ+ individuals in Hong Kong have actively engaged in both individual and collective actions to advocate for their rights and strive for equality (Chan, 2018d, 2019, 2022; Chan & Mak, 2021; Yip & Chan, 2021).

The intersectionality of gender, sexuality, and other identities in shaping LGBTQ+ individuals’ experiences and activism have been highlighted in this domain. For instance, young gay men navigated the interplay between their sexual and cultural/national identities as they engaged in various forms of civic and political participation (Kong, 2019). Older gay men experienced empowerment and social transformation through their involvement in a self-help group formed as a result of a research project (Kong, 2018). Lesbian women’s activism highlighted the significance of labelling practices within their community (Wong, 2016). A genderqueer individual developed genderqueer narratives and virtual representation in gender advocacy (Law, 2021). Pro-LGBT religious groups built an inclusive membership for sexual minorities and contributed to the pursuit of equality (Chan, 2018c). The studies also expanded the scope of LGBTQ+ political and civic participation in Hong Kong by including the experiences of migrant domestic workers and expatriates. LGBTQ+ migrant domestic workers from the Philippines and Indonesia have engaged in activism to address their unique needs and bring queer perspectives into migrant and grassroots movements in Hong Kong (Allmark & Wahyudi, 2019; Lai, 2018). Gay and lesbian expatriates have also played a role in advocating for legal recognition in Hong Kong (Suen, 2021).

The articles also examined various strategies employed by LGBTQ+ activists in their political and civic engagement. Some activists used the human rights discourse to pursue legal gender recognition, while others focused on fostering humanising interactions with transgender individuals to promote acceptance (Madson, 2022). Public attitudes towards LGBTQ+ rights were also examined in this domain. A representative telephone survey conducted in 2016 (*n* = 1008) showed that 32.8% of the public supported same-sex marriage, while 39.4% opposed it (Yeo & Chu, 2018). However, over half of the Hong Kong population supported legal protections against discrimination based on sexual orientation, which challenges the government’s argument against introducing anti-discrimination legislation based on sexual orientation on the ground that there is no ‘majority support’ and that opinion is divided (Suen, 2017).

#### Economic Well-Being

Twelve articles (8%) addressed the economic well-being of LGBTQ+ individuals. The lack of legal protection against workplace discrimination poses serious challenges to the well-being of LGBTQ+ individuals. Around one-third of the respondents (*n* = 792 in total) reported experiencing employment discrimination, and these experiences had a negative impact on their psychological outcomes (Lau & Stotzer, 2011).

In addition to employment, LGBTQ+ individuals in Hong Kong also face discrimination in the rental housing market (Pang et al., 2022; Yung & Lee, 2014). Studies have highlighted the challenges encountered when seeking housing, with discrimination based on sexual orientation and gender identity being prevalent. LGBTQ+ individuals may experience subtle discrimination and micro-aggressions and may be forced to hide their identity (and the existence of a partner/relationship) when attempting to rent a flat (Pang et al., 2022). There is no legal protection against discrimination in the private rental market.

A study of 106 transgender individuals highlighted that more than half (50.9%) had a university degree or higher qualification. However, nearly half (43.4%) had a monthly income below HK$6,000 (about USD$775), which is approximately one-third of the median monthly wage in Hong Kong (HK$19,100, about US$2,440; Census & Statistic Department, 2023). This may reflect the economic struggles experienced by even university educated transgender individuals as a result of discrimination in the employment domain (Suen et al., 2018).

Four articles focused on the influence of social class on gay men (Kong, 2021; Yu, 2018a, b, 2020). These studies examined the unequal access to social, economic, and cultural resources that shapes the challenges and aspirations of local gay men. Class played a pivotal, though often unspoken, role in the everyday life of these gay men, intersecting with age, generation, race, and culture. Another study involving two generations of gay men in Hong Kong underscored the significance of economic independence in constructing their Chinese masculinity (Kong, 2021).

The vast majority of LGB respondents highlighted the importance of legislation against discrimination on the grounds of sexual orientation. Nearly 40% of LGB respondents (*n* = 920) in another study considered emigrating from Hong Kong due to the lack of legal protection against discrimination in public domains (Suen & Chan, 2021).

#### Education

Eleven articles (7%) covered LGBTQ+ individuals’ education, with most focusing on the experiences of LGBTQ+ students in secondary schools. Two articles focused on teachers and social workers in high schools who may encounter LGBTQ+ students (Kwok, 2019; Tang, 2014). The educational sector in Hong Kong presents numerous challenges for LGBTQ+ individuals (Kwok & Kwok, 2022a; Kwok, 2016a, b). There is no anti-discrimination ordinance to protect LGBTQ+ students from individual and institutional discrimination (Kwok, 2016a, 2019). The Court of Final Appeal has ruled that the Basic Law and the Bill of Rights Ordinance, which apply to public schools, provide protection against sexual orientation discrimination. It is important to note, however, that enforcement of these laws is only possible through the court, and applying for judicial review can be complex and expensive.

Schools have not established support programmes for LGBTQ+ students, and some education professionals not only ignore the developmental needs of the students, but also reinforce sexual prejudice when implementing sexuality education curriculums, school practices, and policies (Kwok, 2016a). Harassment based on sexual prejudice is experienced by LGBTQ+ students, and reinforced and exacerbated by school counsellors, teachers, and other adults openly conveying prejudiced messages (Kwok et al., 2012).

Inclusive policies and sexual diversity teacher training to enable schools and teachers to support LGBQ students are nearly non-existent (Kwok, 2019). No mandated programmes have been developed to include LGBQ-related content in teacher training in Hong Kong. Conservative religious beliefs at both the personal and structural levels deter the provision of counselling and mental health services supportive of LGB youth in Hong Kong (Tang, 2014). Teachers and social service providers in schools reflected that they face structural barriers to discussing sexuality in schools (Tang, 2014).

Transgender students face even more significant challenges in the educational sector. The lack of legislative protection and the invisibility of trans-friendly and rights-based sexuality education curricula imply that transgender students in Hong Kong do not have an inclusive and safe space to voice their gender expression and identity (Kwok, 2018). Transgender youth face harassment and discrimination at both the individual and institutional levels in school. They are subjected to strict gender-binary social policies and are forbidden to use chosen names and gender pronouns or wear school uniforms and use bathrooms in line with their gender identity. Many of them have had traumatic experiences at school and face institutional barriers when seeking support from teachers and social workers at school (Kwok & Kwok, 2022b).

#### Personal Security and Violence

Five articles (3%) focused on the personal security of LGBTQ+ individuals. One study examined the experiences of 614 LGB individuals and found that 69.8% of them had encountered violence motivated by prejudice against their sexual orientation, including property damage and verbal, physical, or sexual assault. Verbal violence was the most common, while approximately 10% experienced physical violence (Stotzer & Lau, 2012). Two articles specifically addressed sexual violence among MSM and highlighted their low awareness and perception of the risk of such violence (Choi et al., 2023). MSM who experienced sexual violence reported internal struggles (Choi et al., 2023), altered their behaviour on dating apps, and expressed decreased trust in forming relationships (Choi et al., 2021).

## Discussion

This is the first systematic synthesis of evidence on LGBTQ+ inclusion in Hong Kong of which we are aware; it identifies prevalent exclusion, discrimination, and stigma across major domains of LGBTQ+ peoples’ lives. This includes a legal system that provides very limited, if any, protection and recognition for LGBTQ+ individuals and same-sex relationships (Suen & Chan, 2021; Tang et al., 2020); an unfriendly and sometimes hostile workplace climate where sexual minorities routinely anticipate and experience discrimination (Lau & Stotzer, 2011; Suen & Chan, 2021); an education system that reinforces and exacerbates multiple dimensions of stigma on LGBTQ+ students (Kwok & Kwok, 2022a); unaccepting attitudes from families of origin (Kong, 2020; Tang, 2021); challenges to participating in political and civic development to fight for their rights (Chan, 2019); and an unsafe environment where LGBTQ+ personal security is compromised (Stotzer & Lau, 2012). The pervasiveness of stigma, social exclusion, discrimination, and victimisation experienced by LGBTQ+ individuals in Hong Kong does tangible harm to their well-being, as documented in the substantial literature on LGBTQ+ mental health in Hong Kong (Hu et al., 2022; Mak & Cheung, 2010).

Moreover, findings from this extensive review underscore the multidimensional consequences of the lack of legal protections against discrimination in the workplace, schools, hospitals, and provision of medical and social services (Kwok, 2019; Lau & Stotzer, 2011; Suen & Chan, 2021). The absence of professional and institutional guidelines and trainings, coupled with the absence of legal and policy regulations, renders LGBTQ+ individuals subject to the personal prejudices of a myriad of professionals, managers, colleagues, staff, teachers, and health and social service practitioners otherwise charged with providing competent treatment and support, yet with no means of redress. These findings highlight the vital importance of policy measures—including legal protection against discrimination on the grounds of sexual orientation and gender identity, legal recognition of same-sex relationships, and legal gender recognition—to address the pervasive marginalisation and exclusion of LGBTQ+ individuals in Hong Kong.

### Gaps in the Literature

This scoping review has highlighted significant gaps in the research literature. The vast majority of empirical research (72%) has focused on health, with more than half of the studies (53%) specifically addressing sexual health, particularly HIV transmission and prevention. Although an important concern, this disproportionate focus may be due to the concentration of research funding and scholarly attention on HIV/AIDS-related issues, which has resulted in other domains of LGBTQ+ inclusion, such as education and economic well-being, being understudied. Also, an almost exclusive focus on HIV/AIDS in health-related issues means that research questions concerning other significant aspects of health behaviour and health outcomes are left relatively unexamined among LGBTQ+ individuals in Hong Kong. Moreover, as HIV/AIDS research mainly targets MSM, this disproportionate focus further marginalises women and transgender individuals.

Overwhelming attention on cisgender men has also been observed in this scoping review, with nearly half of the literature (49%) focusing on men, including MSM (36%), gay men (8%), and gay and bisexual men (3%). Only 7% of the articles focused on lesbian (5%) and/or bisexual women (2%). Strikingly, among the 152 articles included, only 2 articles focused on bisexual and pansexual individuals specifically, and 5 articles addressed transgender and genderqueer individuals. The disproportionate distribution of the existing empirical literature reflects an over-representation of gay men and MSM, and a relative under-representation of lesbian and bisexual women, transgender individuals, and genderqueer individuals. This pattern is consistent with findings in LGBTQ+ research literature in other countries (Chakrapani et al., 2023; LaVaccare et al., 2018; Newman et al., 2021). More studies are needed to address the diversity within the LGBTQ+ community and focus on the marginalised experiences of lesbian, bisexual, transgender, and genderqueer individuals.

This scoping review maps out the different terminologies adopted in LGBTQ+ studies over the past 12 years. Among the studies, 37% adopted a behavioural approach to identify their focal population. Apart from the commonly used terminology MSM in HIV/AIDS studies, 1% of the research recruited respondents who have/had same-sex sexual experiences or same-sex partners. A quarter (26%) adopted an identity-based approach and used sexual identity labels such as gay men, lesbian, bisexual men and women, and transgender individuals to identify their research participants, while 23% of the articles addressed LGBTQ+ as a group.

The use of different terminologies across studies has both benefits and limitations for assessing LGBTQ+ inclusion in Hong Kong. Research using different terminologies can capture the multidimensional aspects of desire, behaviour, and identities in LGBTQ+ lives (Richardson, 1983). The use of a behavioural approach to identify the focal population can avoid the complex social and cultural connotations associated with sexual identities. Conversely, the adoption of an identity-based approach can reaffirm the self-determined sexual identity of LGBTQ+ individuals (Young & Meyer, 2005). Employing general terms such as ‘LGBTQ+ ’ and ‘sexual minorities’ can capture a diverse population of individuals with a wide range of desires, behaviours, and identities that do not conform to heterosexuality and/or cisgender norms (Everett, 2013). This is particularly important as there has been an expansion of sexual identities in recent years. More LGBTQ+ individuals may not limit themselves to traditional labels such as ‘gay’, ‘lesbian’, and ‘bisexual’ but adopt more diverse sexual identity labels (Russell et al., 2023). The use of broader terminologies such as ‘sexual minorities’ or ‘LGBTQ+ ’ in research design can ensure a greater degree of inclusion and address the growing diversity within the LGBTQ+ community. However, the employment of different terminologies in studies from 2010 to 2022 makes comparisons across time difficult. One of the primary purposes of the LGBTQ+ inclusion index developed by Badgett and Sell (2018) is to allow comparisons and measure progress towards inclusion across time. Understanding the advantages and disadvantages of different terminologies is key for researchers in conducting future LGBTQ+ research.

Another gap identified in this scoping review is related to the representativeness of the data. Due to the lack of official representative data on the LGBTQ+ population in Hong Kong, the data on LGBTQ+ individuals in the studies included in this scoping review were not representative. Studies on public attitudes that can yield representative data are the only exception to this, as they are based on the sampling framework of the Hong Kong general population provided by the census (Lau et al., 2014a; Suen, 2017; Yeo & Chu, 2017, 2018). One consistent recommendation in global literature on LGBTQ+ inclusion is the importance of collecting official censuses and the availability of representative data on the LGBTQ+ community for researchers, social service providers, and policymakers (Fischer et al., 2022; Herman et al., 2022; Naylor, 2020; Reid et al., 2022). The practice of excluding questions related to sexual orientation and gender identity in government censuses leads LGBTQ+ individuals to be uncounted and rendered more invisible. It also means fewer documented needs, fewer public resources, less political representation, and less visibility in general society (Naylor, 2020). Additionally, the availability of representative data on the LGBTQ+ population can hugely benefit scholarly and policy-focused research to better understand the experiences and needs of these marginalised communities (Fischer et al., 2022; Gates, 2010).

### Expanding the Inclusion Framework

This scoping review suggests expanding the inclusion typology developed by Badgett and Sell (2018) to include a ‘family’ domain, which is in line with emergent findings from other Asian societies such as Thailand (Newman et al., 2021) and India (Chakrapani et al., 2023). The large number of publications (18%) on family, the second largest number after the ‘health’ domain, suggests its relevance and significance to the LGBTQ+ population in Hong Kong. This is likely because family is perceived as the major heteronormative regulatory force for LGBTQ+ individuals in Chinese societies (Kong, 2011). The family domain, including LGBTQ+ individuals’ relationships with their families of origin and their rights to marry and start their own family, is crucial in and of itself and is a determinant for other domains of inclusion. LGBTQ+ individuals experience rejection from their parents and face numerous family pressures to form a heterosexual marriage. The lack of family inclusion was found to have substantial impacts on LGBTQ+ individuals’ overall health, economic well-being, and personal security and safety. The family domain also encompasses policies related to LGBTQ+ inclusion. The lack of legal recognition of same-sex relationships hinders LGBTQ+ individuals from starting their own family, which brings impacts and insecurity to their current lives and future plans. Legal recognition of same-sex relationships has been a key focus of political and civic participation of LGBTQ+ individuals in Hong Kong. To contextualise the inclusion typology in Hong Kong, a Chinese-majority society, consideration of the family domain could provide a more holistic understanding of the current situation of LGBTQ+ inclusion in Hong Kong.

### Strengths and Limitations

This extensive review of the peer-reviewed literature on LGBTQ+ inclusion in Hong Kong forms an important evidence base to evaluate the needs and progress in understanding and acceptance of LGBTQ+ individuals, as well as gaps and directions for research moving forward. However, several limitations should be noted. Given the lack of a previous synthesis of evidence on LGBTQ+ research in Hong Kong, we focused on empirical research and did not include grey literature, such as reports from community-based organisations, media, and popular culture. Furthermore, 50 articles on LGBTQ+ inclusion were identified but excluded from this scoping review because they lacked empirical evidence. Most were published in law journals and provide legal analysis on current policy and judicial review cases (e.g. Barrow & Chia, 2016; Loper, 2011; Wan, 2011); these may contribute to advancing regulations and laws, but were outside the purview of this review. Other articles were primarily cultural analyses of the current social climate, history, traditions, and culture related to LGBTQ+ issues in Hong Kong (e.g. Wong, 2017; Wong & Leung, 2012). Although these papers may contribute insights to the understanding of LGBTQ+ issues, they may be based on assumptions and perspectives informed by taken-for-granted socio-political and cultural norms and anecdotes. Rigorous quantitative and qualitative empirical data are crucial to reflect the lives of LGBTQ+ individuals and to advocate and support progress on LGBTQ+ inclusion in Hong Kong. Future reviews of grey literature, as well as scholarly historical and cultural analyses, may contribute to the breadth and depth of understanding and life experiences of LGBTQ+ individuals in Hong Kong. Additionally, we did not include sources published prior to 2010, given the extensive number of articles reviewed and included, and our primary interest in evidence on the current scenario to inform research, policy, and practice moving forward.

## Conclusion

The literature on LGBTQ+ inclusion in Hong Kong is rapidly expanding, which may reflect growing interest and concern among researchers, policymakers, and the general public. This review provides a comprehensive overview of LGBTQ+ inclusion in Hong Kong and identifies crucial gaps in the literature. It underscores the prevalence and breadth of exclusion experienced by LGBTQ+ individuals in various domains, including education, economic well-being, family, health, political and civic participation, and personal security and violence. This is crucial for informing policy debates, particularly in light of the potential implementation of an alternative legal framework for same-sex partnerships in Hong Kong. The review also demonstrates the adoption of Badgett and Sell’s (2018) LGBTQ+ inclusion index framework beyond the Global North and expands the index to include the ‘family’ domain. Future studies should address the gaps identified by this scoping review. Specifically, more research should focus on underrepresented groups, including lesbian, bisexual, transgender, and genderqueer individuals. Researchers should also carefully consider the terminology adopted for their studies. This review also highlights the importance of collecting empirical data and obtaining representative data on the LGBTQ+ population to assess the current situation and progress made in LGBTQ+ inclusion in Hong Kong, as well as to inform policy changes related to LGBTQ+ rights.

## Data Availability

N.A.

## Code availability

NA.
